# Designing a reference architecture for health information systems

**DOI:** 10.1186/s12911-021-01570-2

**Published:** 2021-07-08

**Authors:** Joep Tummers, Hilde Tobi, Cagatay Catal, Bedir Tekinerdogan

**Affiliations:** 1 Information technology, Wageningen University & Research, Hollandseweg 1, 6701KN Wageningen, The Netherlands; 2 Biometris, Wageningen University & Research, Droevendaalsesteeg 1, 6706OB Wageningen, The Netherlands; 3 Department of Computer Science and Engineering, Qatar University, 2713 Doha, Qatar

**Keywords:** Electronic patient dossier, Reference architecture, Software architecture, Health information systems, Unified modeling language

## Abstract

**Background:**

Healthcare relies on health information systems (HISs) to support the care and receive reimbursement for the care provided. Healthcare providers experience many problems with their HISs due to improper architecture design. To support the design of a proper HIS architecture, a reference architecture (RA) can be used that meets the various stakeholder concerns of HISs. Therefore, the objective of this study is to develop and analyze an RA following well-established architecture design methods.

**Methods:**

Domain analysis was performed to scope and model the domain of HISs. For the architecture design, we applied the views and beyond approach and designed the RA’s views based on the stakeholders and features from the domain analysis. We evaluated the RA with a case study.

**Results:**

We derived the following four architecture views for HISs: The context diagram, decomposition view, layered view, and deployment view. Each view shows the architecture of the HIS from a different angle, suitable for various stakeholders. Based on a Japanese hospital information system study, we applied the RA and derived the application architecture.

**Conclusion:**

We demonstrated that the methods of the software architecture design community could be used in the healthcare domain effectively and showed the applicability of the RA.

**Supplementary Information:**

The online version contains supplementary material available at 10.1186/s12911-021-01570-2.

## Background

Healthcare relies on health information systems (HISs) to support various care processes and receive reimbursement for the care provided. Examples of functionalities are financial management, daily reporting, and medication management [1–3]. Unfortunately, current HISs still have some drawbacks. For example, lack of interoperability resulting in care professionals having difficulty communicating files [4, 5]. Other studies on HISs reported problems with poor interface design [6, 7], poor security [8, 9], missing features [10, 11], lack of professional support [12, 13], limited use [6, 14], and low data quality [1, 15]. Most of these problems occur when relevant standards, procedures, and guidelines are not followed effectively.

Because HISs consist of many interrelated software modules that should communicate, coordinate, and evolve over time [16], the software architecture is critical in HIS design. Bass et al. [17] define the software architecture of a program or a computing system as: “The structure of the system, which comprises software elements, the externally visible properties of those elements, and the relationships among them.” The software architecture supports communication on the system, guides design decisions, informs maintenance, and facilitates architectural analysis of the overall system [18].

There are two main approaches for software architecture design: informal and formal. The back-draw of informal software architecture design relying on boxes-and-lines models, is that such a representation of the system is hard to understand because it is not standardized and does not follow a particular language. The formal approach follows the well-established ISO/ISEC/IEEE 42010 standard [19], which ensures unambiguous communication.

A particular type of architecture that is generic and can help design specific software architectures for multiple software systems is the Reference Architecture (RA). An RA is a generic design that can be used to derive specific Application Architecture (AAs) based on the identified stakeholders’ concerns, more quickly and with higher quality [20, 21]. The RA serves as an architecture blueprint for future software architects and should provide a standardized lexicon, taxonomy, and (architectural) vision [21, 22] . In the (grey) literature, several RA designs have been proposed for HISs [23–30]. More information on these RAs is available in the Related Work Section.

In practice, the derivation of the AAs from RAs is not trivial for two basic reasons. First of all, some of the proposed RAs do not focus on HIS in general, but only address the hospital sub-domain [29, 31]. Secondly, the proposed RAs do not seem to follow a proper architecture documentation guideline. [26–28, 30]. Furthermore, these RAs are far from complete, which hampers the design of the required AAs.

The problems stakeholders experience with HISs require more clarity in healthcare’s complex digital landscape, a clarity that RA provides. Therefore, the objective of this article is to develop an RA for HISs following well-established architecture design methods. The RA is dedicated to the healthcare domain and is represented using the software architecture viewpoints. To illustrate and evaluate the RA, an AA was derived in a case study on a Japanese hospital. The paper concludes with lessons learned and a discussion of the proposed RA.

## Methods

### Research questions

The following research questions were identified:

- RQ1: What are the stakeholders and their concerns related to HISs?
- RQ2: What is a feasible Reference Architecture for HISs?
- RQ3: Does the Reference Architecture allow for the derivation of a specific Application Architecture?

**Fig. 1:**
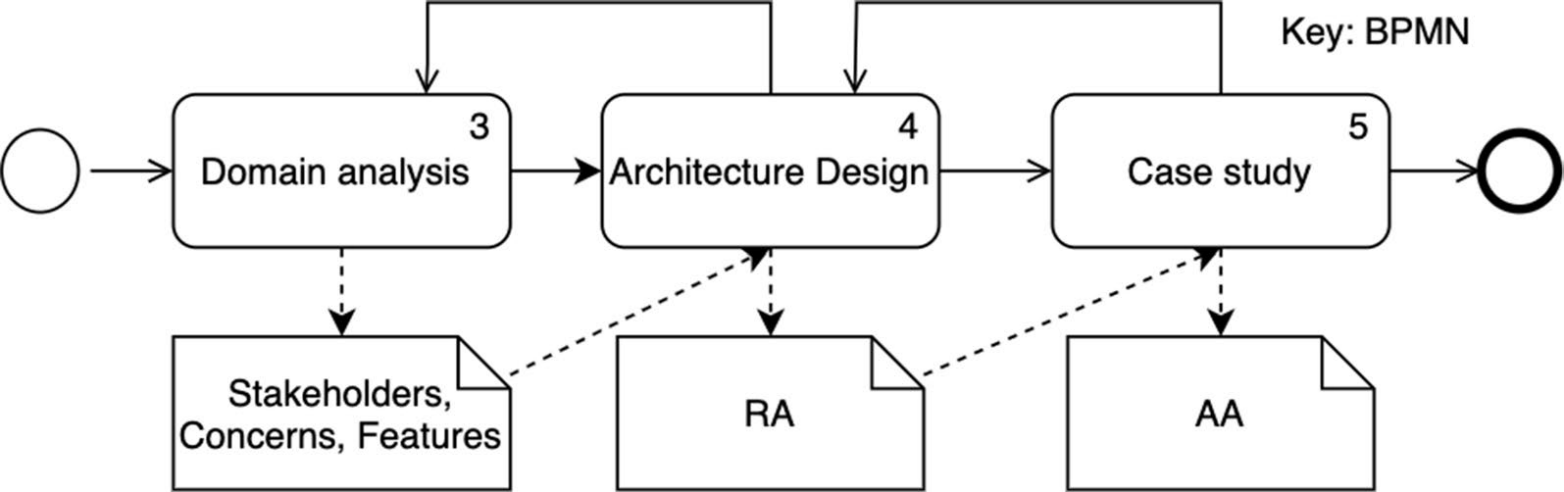
The adopted approach for the RA design. Numbers inside tasks represent corresponding section numbers

Our approach to these questions is depicted in Fig. 1. Domain analysis is defined as the systematic activity for deriving and storing domain knowledge to support the engineering design process [32]. Domain analysis consists of domain scoping and domain modeling. Domain scoping identifies the domain’s scope and the necessary knowledge sources to derive the key concepts [33, 34]. Domain modeling aims at representing the domain knowledge in a reusable format.

Based on the domain analysis, we choose the relevant viewpoints [35] for our architecture design step. We continued with a case study, to evaluate the RA’s suitability for deriving an AA.

### Method for deriving and evaluating application architecture

The RA can be used as a starting point for creating an AA [20]. The AA is described in this study as the software model of a specific application displayed through a combination of architectural views. To begin, an RA was created.

**Fig. 2:**
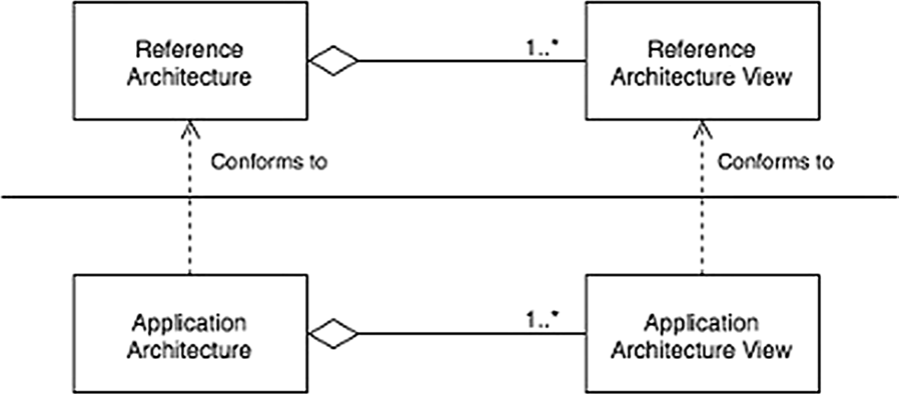
Methods used for deriving the AA. Each view from the RA will lead to a view in the AA, Adopted from [36]

**Fig. 3:**
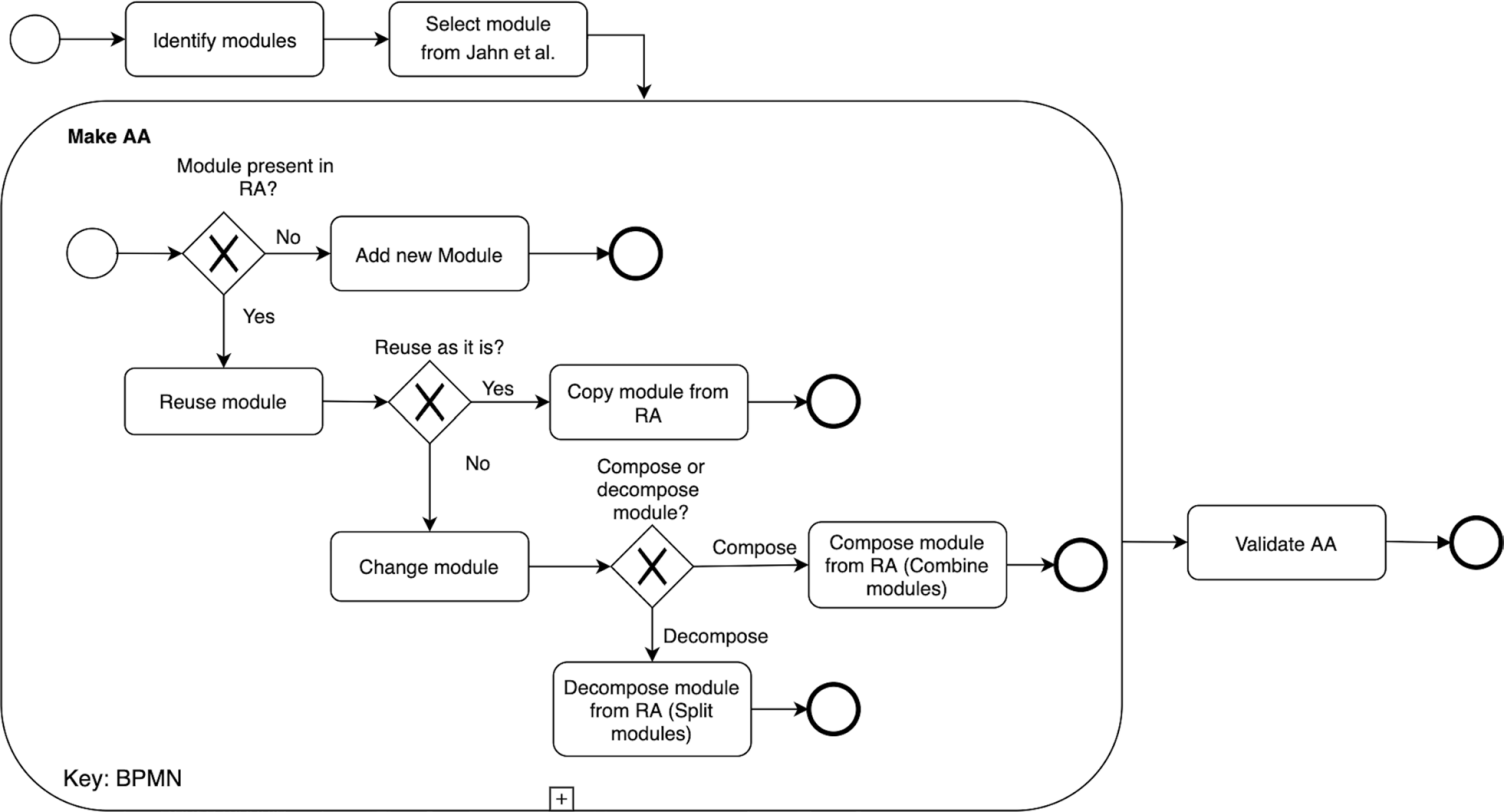
Approach followed in building AA from RA Adapted from Tummers et al. [36]

The view of the RA was used to generate the corresponding view of the AA, as seen in Fig. 2. Figure 3 depicts the procedure followed for this derivation. For each view of the reference architecture, this approach was used; the application’s necessary entities were first listed; then entities from the corresponding RA view were chosen based on the entity from the application. The required entity is reused if it could be identified in the RA; otherwise, a new entity was introduced to the AA. If the entity is located in the RA, it was examined to see if it can be reused in its original state or whether it has to be changed. If the names of the modules were the same or if the modules were interchangeable (e.g., financial management vs. economic management [37]), the modules were considered entirely reusable. The module may be composed or decomposed if it is not reusable in its present state and thus had to be modified. In a composition, multiple RA modules were merged into a single AA module. As an example of a composition, a data transfer module and data collection module could be merged into a data processing module (see [38]). After the decomposition, an RA module is broken down into several smaller modules in the AA. Finally, the reusability of the RA’s entities was explored and the RA’s usability (to derive the AA) considered. Concluding, making an AA for a particular settings (i.e. case study), serves as validation for the RA [20, 36].

This approach as described above and depicted in Fig. 3 , has been used in a variety of other domains such as agriculture [36], supply chains [39], and smart warehouses [33].

## Results

### Domain analysis

To scope and model the domain, we performed a systematic literature review [40] to identify papers in which HISs, their domains, stakeholders and, concerns and features were described. This resulted in a set of 11 papers [7, 10, 41–49].

#### Domain scoping

HISs cover a wide range of sub-domains within healthcare. Many HIS papers focus on the hospital sub-domain [7, 42, 47], others focus on the primary care [10], pediatrics [43], outpatient care [45, 47], and diabetes care [49]. The most common stakeholders and their concerns for HISs development are presented in Table 1. While some stakeholders are generic for HISs, such as the patient, other stakeholders are more domain-specific, such as the Laboratory.

**Table 1:** Key stakeholders (in alphabetical order) and their main concerns

Role	Concerns
Administrative staff	Wants easy data entering and retrieval
Automated data source	A protocol to safely upload data from heart rate monitor, wearable technology, medical robots, et cetera
Care professional	Wants system to be easy to use such that information can be quickly entered, retrieved, and shared
Government	Wants the system to comply with all their regulatory standards
Healthcare manager	Needs system to provide overviews and reports
HIS developer	Develops system in time within the planned budget
Insurance company	Wants compatibility with their system for reimbursement
Laboratory	Wants compatibility with their measurement devices
Other HIS	Needs to be able to communicate with HIS and exchange data
Patient and/or representative	Wants data to be stored safe and secure. Wants care professionals to have the right information at the right time. Wants reimbursement of care
Pharmacist	Needs medication management to be an integral part of the system
Plug-in developer	Wants easy to use platform for plug-in development
Research institute	Needs system to provide structured data such that it can be used for research
Secretary	Needs system for making appointments and administrative tasks
HIS administrator	Wants system that is easy to maintain and adequately documented

#### Domain modeling

**Fig. 4:**
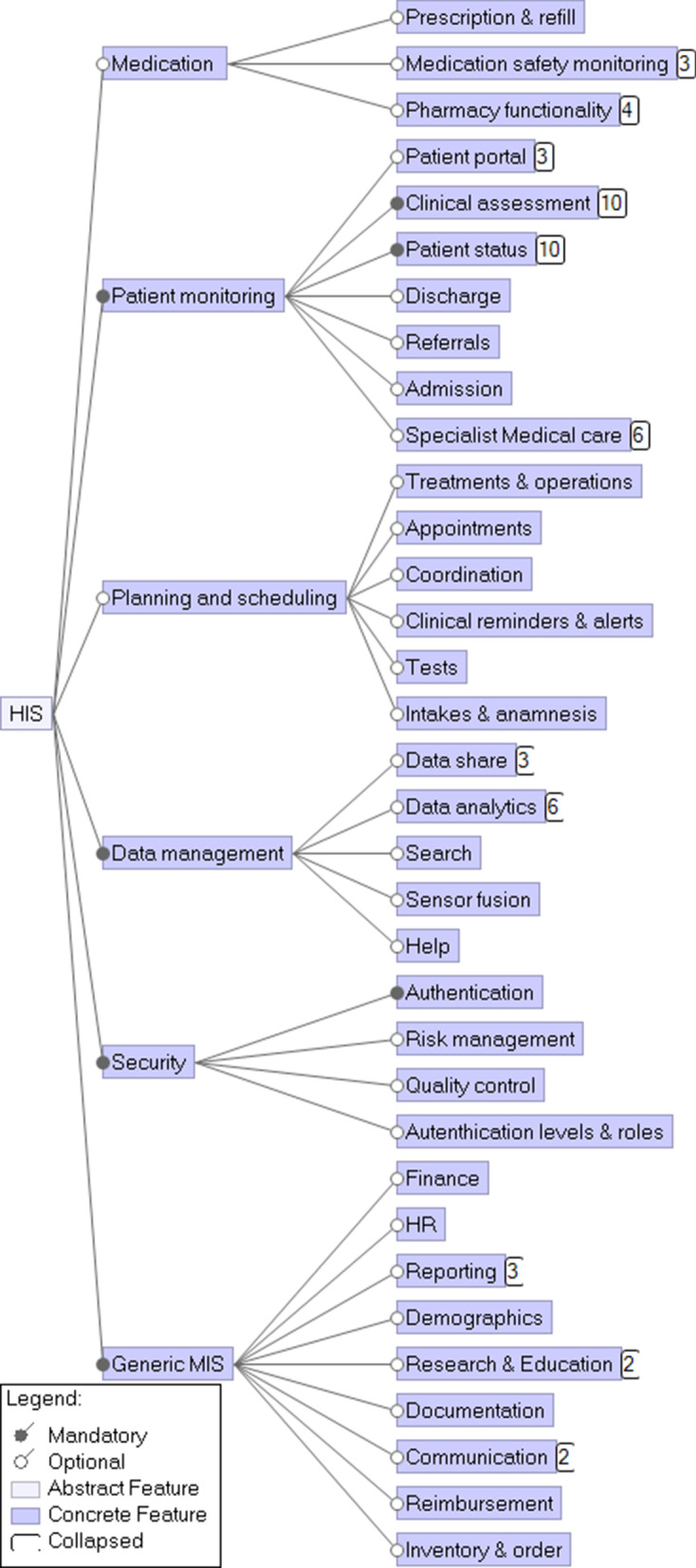
A downsized version of the Feature model for HISs. Numbers on the right-hand side of the features represent the number of sub-features not shown

To model the features of the HIS domain, feature modeling was adopted. A feature model represents the domain knowledge and desired system by distinguishing common, alternative, and optional(e.g., sub-domain specific) features of the system, and the interdependencies amongst these features [50]. The feature is defined as “a prominent or distinctive user-visible aspect, quality, or characteristic of a software system or system” [51]. Sub-features of a more general feature are shown under the most general feature in a tree-shaped model [51]. Our feature model for the HIS is presented in Fig. 4 and is based on the features mentioned in the literature.

We split the full set of features into six main features (Middle column of Fig. 4). The Generic Management Information System (MIS) feature contains non-domain-specific features. The Data management feature contains features related to the management of data and data-driven decision-making by care professionals. Medication management and Patient monitoring are typical HIS features. Planning & scheduling is a feature mainly used by secretaries and administrative staff. Last, but certainly not least, the Security feature must ensure the system’s resilience and protection of its data.

### Architecture design

In the next section, the selected viewpoints were used for designing the RA are described. In the subsequent sections, the HIS RA views were built from these four viewpoints.

#### Selection of views

Although the HIS’s main purpose is to assist in the current daily operations, it should also be flexible and adaptable to facilitate different long-term visions [16, 52]. To do so, the RA needs to cover all features of the feature diagram in Fig. 4. The RA should also cater to users in all different sub-domains of healthcare and facilitate tailoring to local needs. After all, a hospital HIS needs to meet different demands than a general practitioner’s HIS and thus, will have different architectural decompositions.

For modeling the RA, we adopted the Views & Beyond (V&B) approach [35]. This approach consists of selecting out of 17 predefined viewpoints the ones of interest to certain stakeholders. The four viewpoints of particular interest to key stakeholders in the healthcare domain selected for modeling the HIS RA are the context diagram, decomposition view, layered view, and deployment view.

#### Context diagram

**Fig. 5:**
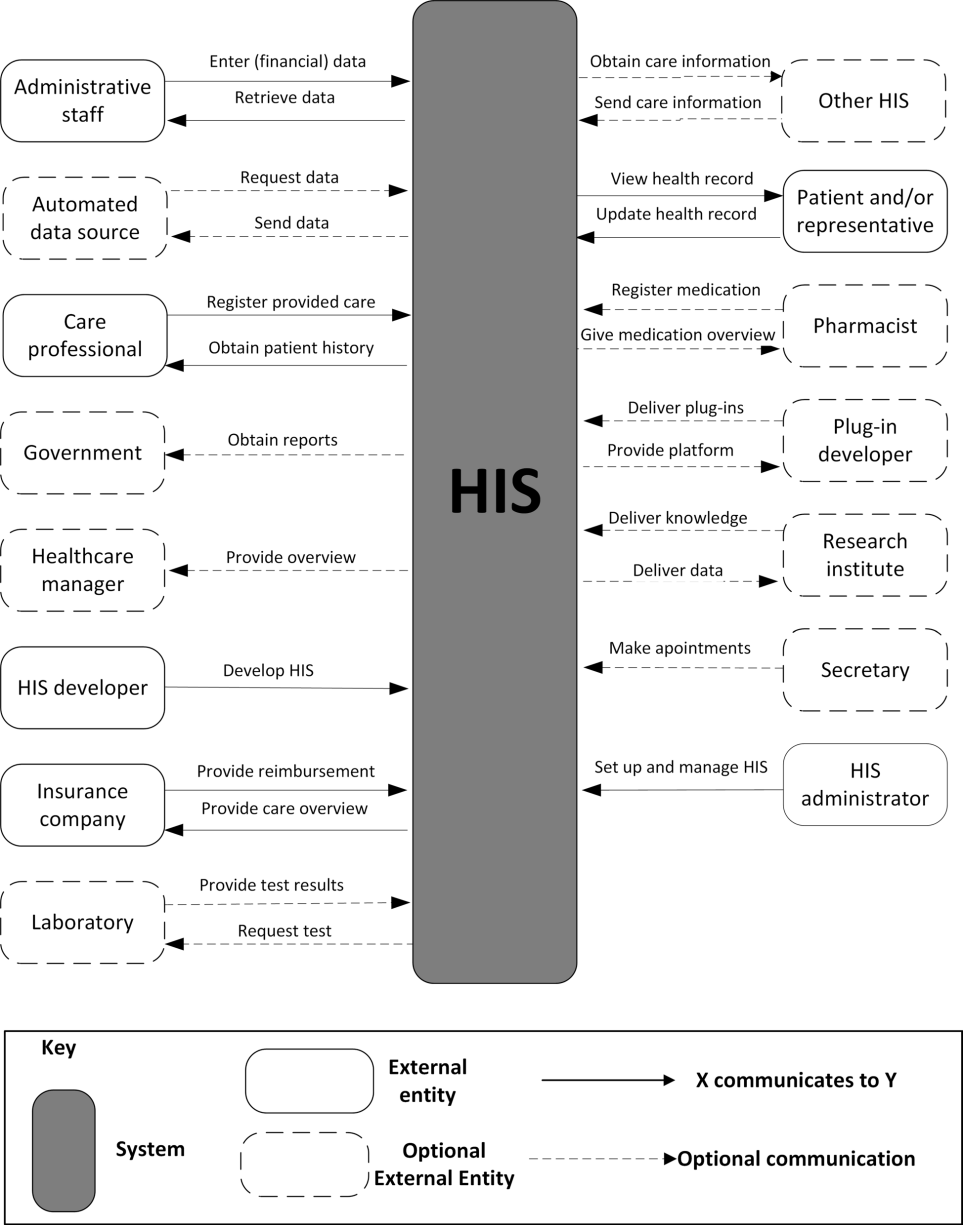
The reference context diagram. Only the interactions considered the most important are shown

The context view of a system contains the entities that are outside the system’s scope but have a direct relation with the system [53]. The context diagram represents the context view and shows the system boundaries, environment, and the entities it communicates with [54]. The reference context diagram for the HIS is presented in Fig. 5.

The external entities and their communications with the HIS were based on the stakeholders and their concerns from Table 1. Six external entities are considered obligatory: the HIS cannot function without them. The optional entities can be absent in simpler HISs such as automated data sources or are (sub)domain-specific, such as the laboratory. Besides, some (sub)domains may require specific entities that are not shown in the reference context diagram. Many entities have two-way communication with the HIS, meaning that the HIS communicates with the entity and vice versa. External entities with a one-way communication with the HIS, are rarer. For example, a governmental organization can receive reports from the HIS, but this organization has no authorization to access the HIS data. We only describe one type of communication per interaction due to space limitations, in practice, there are many more possibilities.

#### Decomposition view

**Fig. 6:**
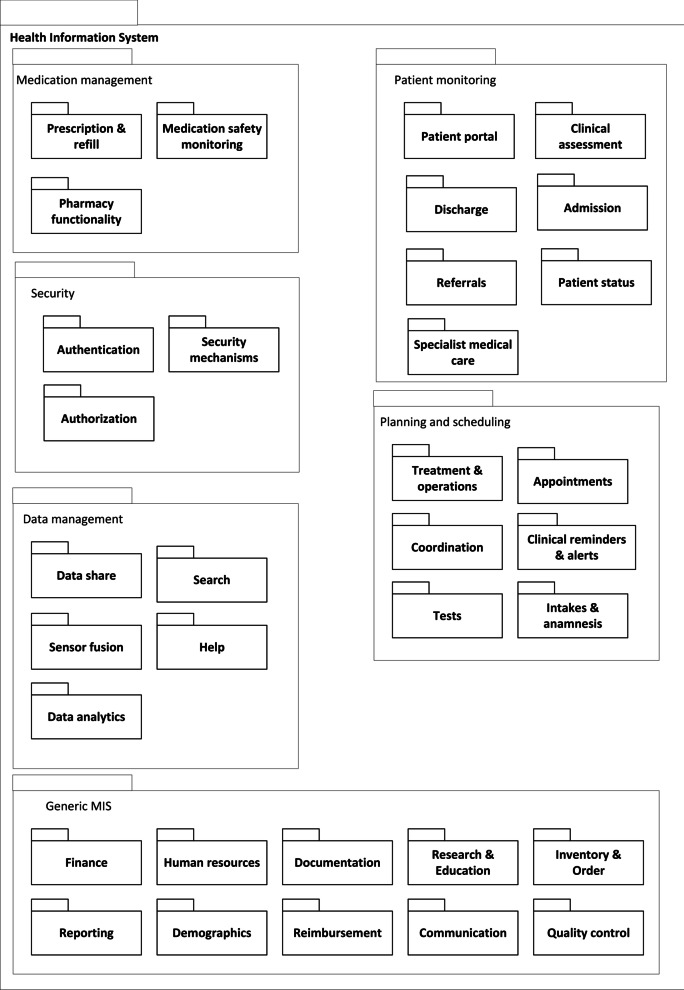
The reference decomposition view of the HIS

The decomposition shows how a system can be decomposed into multiple (sub)modules and how they relate to one another (parent-child). This view often is the basis for HIS design, development, and system documentation [55]. The decomposition view helps to check for the presence of the required modules for all stakeholders. The HIS RA decomposition view consists of six modules with 34 sub-modules, see Fig. 6.

The first module is Medication management, containing sub-modules related to medication handling, distribution, and safety monitoring. The second module is Patient monitoring and contains sub-modules related to the assessment, admission, discharge, status, and referrals of patients, and is input for the electronic health record. The Patient monitoring module also contains a sub-module labeled Patient portal in which the patient can check his/her files. The Security module with the sub-modules Authentication, Authorization, and Security mechanisms must ensure the privacy and security of the HIS and its data. Module number four is Planning and scheduling, with sub-modules used by various stakeholders to ensure proper care coordination. The Generic MIS module is not healthcare specific. Its sub-modules are important to keep track of assets, such as staff and inventory, to provide means for organization-wide communication, quality control, and financial affairs. Often the features from the generic MIS module can be found in so-called enterprise resource planning (ERP) systems. These systems are business management system solutions which are used for managing, automating, and integrating all the business functions within an organization [56–58]. These five modules generate data, which needs management. This happens in the Data management module, which has sub-modules to ensure proper import, sharing, analysis, and data search.

Figure 6 shows all described modules and sub-modules of the RA for HIS. A specific Application Architecture (AA) consists of a selection of these modules tailored to the stakeholders’ requirements.

#### Layered view

**Fig. 7:**
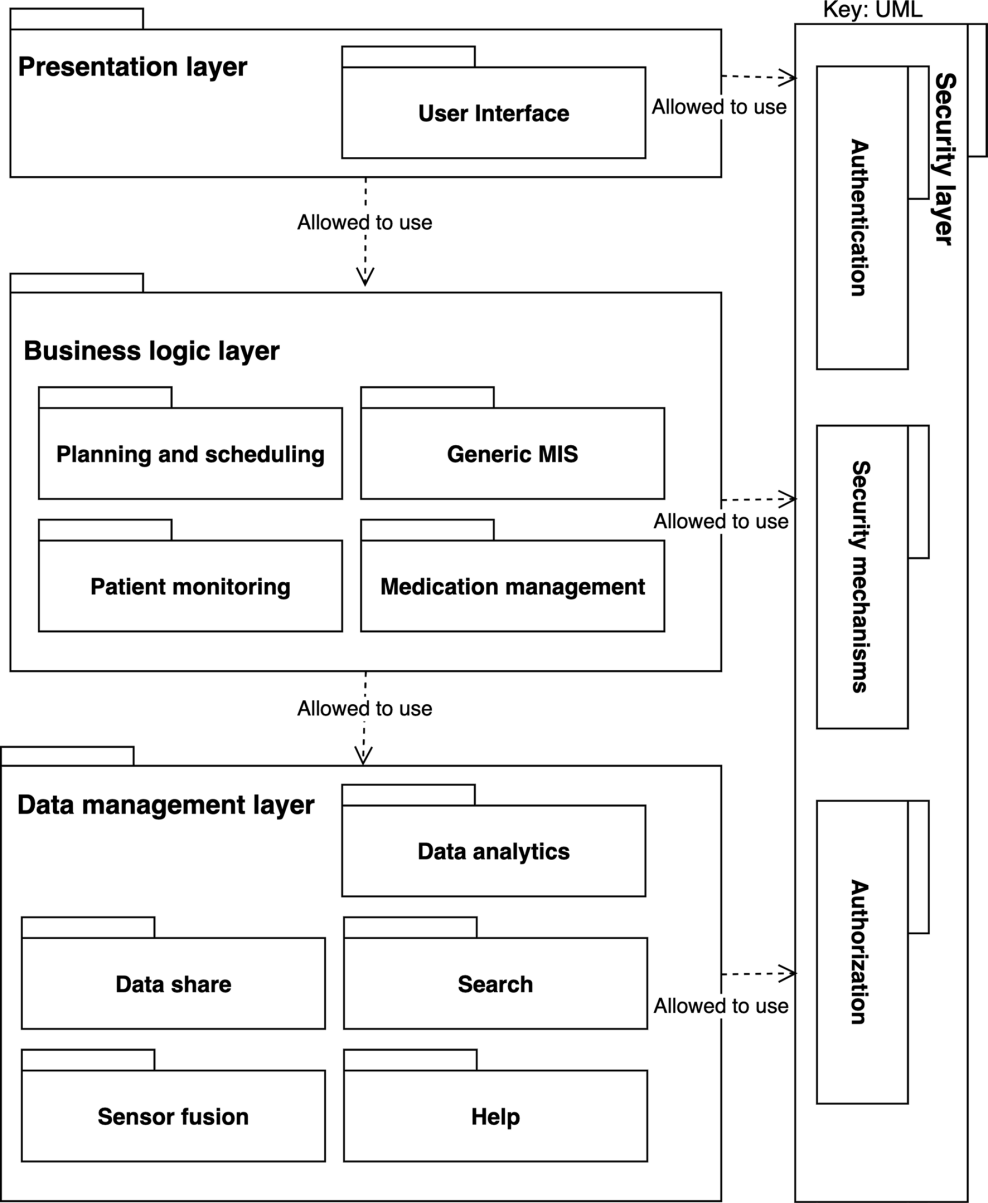
The reference layered view for the HIS

The layered view reflects the software modules’ allocation into different layers, based on a unidirectional “allowed to use” relationship between the layers Clements et al. [35]. We decided to base our layered view on the standard of enterprise software systems because of its flexibility (Fig. 7). Starting at the top, the layered view consists of a presentation layer with a User Interface (UI). The presentation layer relies on the business logic layer that determines how data are created, stored, and processed. The business logic layer contains the Planning and scheduling, Generic MIS, Patient monitoring, and Medication management modules from the decomposition view (Fig. 6). These four modules, the backbone of any HIS, generate and use data from the Data management layer. The Data management layer contains sub-modules to simplify access to the data. To provide overall HIS security, a vertical layer connected to all three horizontal layers was added. This Security layer contains the modules: Authentication, Security mechanisms, and Authorization for safety and security at all system layers.

#### Deployment view

In the deployment view, software modules are allocated to the hardware entities on which they are executed. This view is useful for analyzing the performance, availability, reliability, and security aspects of the system [35]. Due to the vast diversity of HISs, we decided to develop a generic deployment view that can represent many of HISs across care domains

**Fig. 8:**
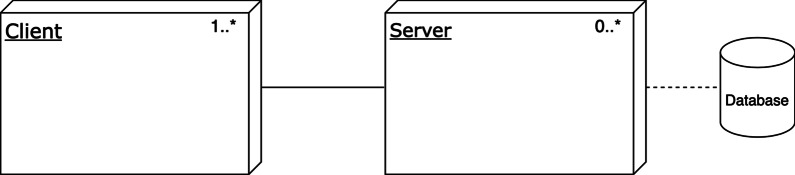
The reference deployment view of the HIS

The deployment view (Fig. 8) shows one or more clients and zero or more servers. If there is a client only, and no server, the deployment is a standalone desktop application or a thick-client with all modules on the client-side. A client-server application consists of at least one server and multiple clients, for example, thin clients with modules located on one or multiple servers. Finally, a system with multiple clients and multiple servers that communicate using cloud computing technology is cloud-based.

A system will most likely have a back-up server in case the original server goes down, but a combination of other types of servers is also possible. These other types of servers could include load balancing servers to allow for big data analytics, as well as application servers, web servers, and database servers. The RA deployment view also provides space for a web-based application. In that case, only an internet browser is required on the client-side with which the end-user can use the HIS. The server is often provided by the software supplier, which contains the modules to host the web page and store the data.

Depending on the specific requirements the allocation of the modules as identified in the decomposition view can be allocated in various different ways over the selected nodes in the deployment view.

### Case study Chiba University Hospital

This study’s primary objective was to propose and evaluate the RA. We decided to base the illustration and evaluation on a case study from the literature, as no site visits were possible due to the COVID-19 pandemic. For our case study, we used the well-detailed article by Jahn et al. [59] in which they compare a Japanese and German hospital HIS using the three-layer graph-based meta-model ($3\hbox {LGM}^{2}$) [31]. When presented and inspected visually, the $3\hbox {LGM}^{2}$ model combines the UML decomposition view, uses view, and layered view.

Our case study was done by developing an AA. for the Japanese Chiba University Hospital (CUH) based on Jahn et al. [59]. Figure 3 shows the approach followed to build the AA.

#### Feature diagram

The 104 modules of the CUH model in Jahn et al. [59] (page 6 Fig. 5) were mapped onto the features from our feature module (Fig. 4). We added 47 sub-features to meet the level of detail presented in Jahn et al. [59]. Interestingly, Jahn et al. [59] listed more detailed Patient monitoring and Generic MISs features, which we included as sub-features in Additional file 1: Fig. 1. In contrast, our RA was more detailed concerning the other HIS features.

#### Context diagram

Although the stakeholders of the HISs are not explicitly mentioned in Jahn et al. [59], we were able to make the application context diagram based on mentioned systems such as a Laboratory Information Systems and a Pharmacy Department System. The stakeholders and other entities of such systems combined with the obligatory entities and interactions from Fig. 5, provided the application context diagram. There was no need to add extra external entities (see Fig. 2 in the Additional file 1). We used 12 out of 15 (80%) entities from the RA context diagram.

#### Decomposition view

The decomposition view extracted from Jahn et al. [59] is presented in Additional file 1: Fig. 3. Despite slightly different wording in the labels of (sub)modules, we could make the decomposition view, which listed 104 modules from the feature model. Seven sub-modules from our RA were not found in the Japanese HIS and removed from the application decomposition view. Therefore, we utilized 27 out of 34 modules from the RA decomposition view, resulting in a re-use of 79%.

#### Layered view

Although the authors used the term “layers” differently than we do, the provided information allowed us to derive the layered view using our own design choices. The result was the same as depicted in Fig. 7 above.

#### Deployment view

**Fig. 9:**
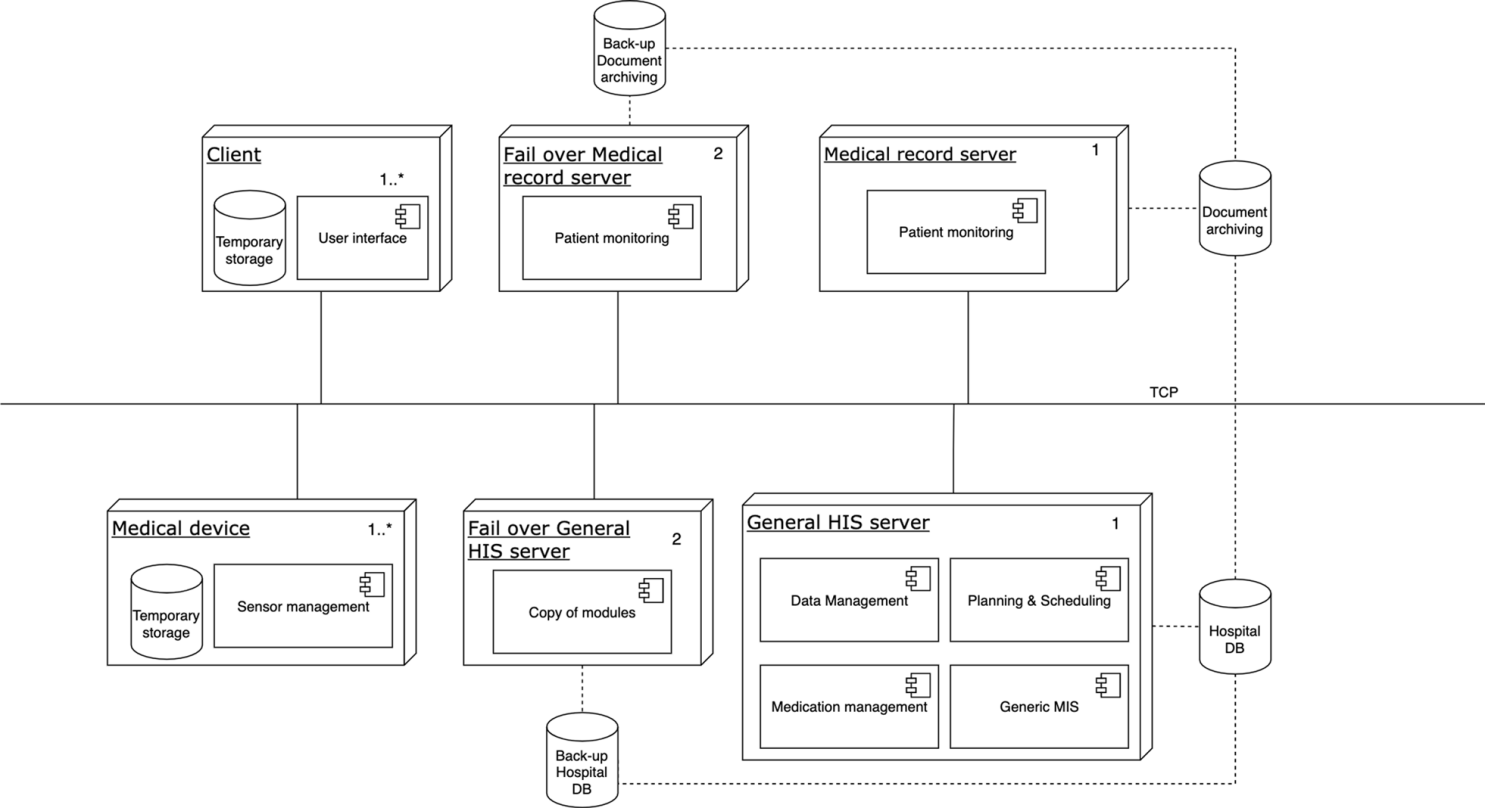
Deployment view for the Chiba University Hospital based on Jahn et al. [59]

Jahn et al. [59] provide limited information about the CUH HIS deployment, but it does show the CUH databases. From this information, we inferred the deployment situation at the hospital. The deployment view is available in Fig. 9.

As discussed above and shown in the Additional file 1, we could successfully derive an AA for the CUH case from our RA. Making the views for the case study took us about two days (16 h). Based on this case study, we made some minor changes to our RA, which were already included in Figs. 4, 6, and 7.

## Discussion

To the best of our knowledge, this is the first RA for the health care domain built using standard architecture design approaches from the software architecture community. In this discussion, we critically reflect on our results and compare this study with related work.

### Critical reflection on the results

For the domain analysis, we relied on scientific articles. A more extended data collection from grey literature or expert interviews might have yielded different input for the viewpoint selection. We believe that the scientific articles provided a factual basis for the viewpoints because of their diversity across care domains, and, indeed, our case study did not suggest otherwise.

Based on the domain analysis, we identified 15 key stakeholders for HISs because of their relevance to almost all HISs. The domain was modeled with the feature diagram, which provided a broad overview of the different features demanded for HISs. The feature diagram included the most relevant features and, when needed could be extended with additional (sub)features, as illustrated in the case study section. This allows the feature model to evolve with the changing health care domain.

Based on the key stakeholders’ concerns and input from the domain analysis, four viewpoints were selected to model the RA. Together, these viewpoints gave a broad and solid overview of HISs. The Context Diagram and Decomposition View showed the architecture from the stakeholders’ perspective, the Layered and Deployment View provided a standardized technical representation of HISs. The Deployment View (Fig. 8) was modeled generically to allow for various deployment alternatives, as illustrated in the case study (Fig. 9).

In current practice, the modules described in the decomposition view are often implemented by a combination of systems. In a hospital, for example, a hospital information system, an order management system, a pharmacy information systems, and many more systems are used. At first sight, a fully integrated ERP system would be an option to align these processes and systems. However, there are several difficulties in using ERP systems in the healthcare sector. First of all, the alignment of business processes with the ERP system is not an easy task, and the success of the project, therefore, depends on the complexity of the processes in the environment. For this alignment, either the processes or the ERP system have to be adapted, but some ERP systems require a lot of effort to be adapted to the required processes. Another problem is related to the vendor lock-in problem [60]. When an ERP system is adapted for the healthcare provider, there is too much dependency on the vendor and the consultants who can provide the required services.

The case study was based on a peer-reviewed article due to the COVID-19 pandemic. Although Jahn et al. [59] did not explicitly name stakeholders, the paper contained sufficient detail to derive the context diagram and the decomposition view. Similarly, we were able to derive the layered and deployment view based on the detailed information Jahn et al. [59] provided. The use of four views to derive the AA was demonstrated. In theory, the same procedure can be used to generate other potential perspectives (e.g. use views, layered views etc.). To do so, the appropriate views must be defined based on the chosen system’s particular application requirements [35] . Till all the necessary views have been determined, the approach described above will be followed; that is, reference views will be established first, followed by application views. When using this reference architecture for the development of a new system, it is very important to make use of the different standards for HISs. In order to ensure interoperability with other systems, standards such as HL7 FHIR should be used [61] Furthermore, the diagnoses in the systems should also be standardized using codes such as ICPC-2 or ICD11 [62, 63].

Future work will expand towards cases in the long-term care domain to further demonstrate our RA’s applicability.

### Related work

Several other RAs for HISs have been published. The pioneer RICHE RA from 1993 [23] has an open architecture with three layers: user applications, basic applications, and information systems. Despite its old age, the paper described many problems that have remained unsolved up until today. Wartena et al. [24] described in 2010 a RA for a personal telehealth ecosystem with a focus on networking and communication, ignoring other features.

More RAs for HISs are found in grey literature, such as white papers and technical reports. These RAs are often characterized by none [25] or some diagrams only [26–28], and do not apply any formal software architecture modeling technique, as defined in the computer science literature [64].

We found three papers that used diagrams systematically to describe their RA for hospitals [29, 31], and healthcare in general [30]. Nictiz [29] presented an RA for hospitals using an Archimate model [65]. Their RA showed similarities with ours: their domain ‘*reference model*’ contained many elements from our decomposition view and layered view. However, the Archimate Model is limited to the scope of enterprise modeling [66] and is based on the by now replaced IEEE 1471 standard [67]. In contrast, UML has a much broader scope and contains many more modeling concepts to choose from, 150 instead of 50. Winter et al. [31] based their RA for the hospital domain on the UML-based 3LGM^2^ model, which had also been used by Jahn et al. [59]. Winter and colleagues’ metamodel for modeling Hospital Information systems, shows similarities with our RA as explained in Section 5. An RA with a similar scope to ours is ATOS’ “IT Reference Architecture for Healthcare” [30]. They did not use UML models, but an informal approach to display the ICT services for HIS development. Their RA shows some overlap with our decomposition view but ignores a deployment view.

Compared to the other RAs, our RA is generic, uses UML models, and addresses the entire healthcare domain.

## Conclusions

In this study, we showed that the methods of the software architecture design community could be used in the healthcare domain effectively: we proposed a generic RA for HISs. We have shown the suitability of the RA for deriving the AA for a University hospital in Japan. Our method of evaluating an RA was successful for one case study. In our future work, we will use this method to a greater extent and apply the reference architecture for designing the architecture of various other HISs.

## Supplementary Information

**Additional file 1**. Application architecture figures.

## Data Availability

Not applicable.

## Abbreviations

HIS: Health information system
RA: Reference architecture
AA: Application architecture
MIS: Management information system
UML: Unified modelling language
V&B: Views and beyond
UI: User interface
3LGM^2^: Three layer graphbased metamodel
CUH: Chiba University Hospital

## References

[1] Kihuba E, Gathara D, Mwinga S, Mulaku M, Kosgei R, Mogoa W, Nyamai R, English M (2014). Assessing the ability of health information systems in hospitals to support evidence-informed decisions in Kenya. Global Health Action.

[2] Adler-Milstein J, Holmgren AJ, Kralovec P, Worzala C, Searcy T, Patel V (2017). Electronic health record adoption in US hospitals: the emergence of a digital “advanced use” divide. J Am Med Inform Assoc.

[3] Bhatti UA, Huang M, Wu D, Zhang Y, Mehmood A, Han H (2019). Recommendation system using feature extraction and pattern recognition in clinical care systems. Enterprise Inform Syst.

[4] Goh WP, Tao X, Zhang J, Yong J (2016). Decision support systems for adoption in dental clinics: a survey. Knowl-Based Syst.

[5] O’malley AS, Draper K, Gourevitch R, Cross DA, Scholle SH (2015). Electronic health records and support for primary care teamwork. J Am Med Inform Assoc.

[6] Friedman A, Crosson JC, Howard J, Clark EC, Pellerano M, Karsh B-T, Crabtree B, Jaén CR, Cohen DJ (2014). A typology of electronic health record workarounds in small-to-medium size primary care practices. J Am Med Inform Assoc.

[7] Pickering BW, Dong Y, Ahmed A, Giri J, Kilickaya O, Gupta A, Gajic O, Herasevich V (2015). The implementation of clinician designed, human-centered electronic medical record viewer in the intensive care unit: a pilot step-wedge cluster randomized trial. Int J Med Inform.

[8] Khajouei R, Gohari SH, Mirzaee M (2018). Comparison of two heuristic evaluation methods for evaluating the usability of health information systems. J Biomed Inform.

[9] Park Y-T, Han D (2017). Current status of electronic medical record systems in hospitals and clinics in Korea. Healthcare Inform Res.

[10] Paré G, Raymond L, de Guinea AO, Poba-Nzaou P, Trudel M-C, Marsan J, Micheneau T (2015). Electronic health record usage behaviors in primary care medical practices: a survey of family physicians in Canada. Int J Med Inform.

[11] Whitt KJ, Eden L, Merrill KC, Hughes M (2017). Nursing student experiences regarding safe use of electronic health records: a pilot study of the Safety and Assurance Factors for EHR Resilience guides. Comput Inform Nurs.

[12] Grout RW, Cheng ER, Carroll AE, Bauer NS, Downs SM (2018). A six-year repeated evaluation of computerized clinical decision support system user acceptability. Int J Med Inform.

[13] Kaipio J, Lääveri T, Hyppönen H, Vainiomäki S, Reponen J, Kushniruk A, Borycki E, Vänskä J (2017). Usability problems do not heal by themselves: national survey on physicians’ experiences with EHRs in Finland. Int J Med Inform.

[14] Vossebeld DM, Puik ECN, Jaspers JEN, Schuurmans MJ (2019). Development process of a mobile electronic medical record for nurses: a single case study. BMC Med Inform Decis Mak.

[15] Dunsmuir DT, Payne BA, Cloete G, Petersen CL, Görges M, Lim J, von Dadelszen P, Dumont GA, Ansermino JM (2014). Development of mHealth applications for pre-eclampsia triage. IEEE J Biomed Health Inform.

[16] Locatelli P, Restifo N, Gastaldi L, Corso M. Health care information systems: architectural models and governance. Innov Informa Syst Modell Tech. 2012;71–96.

[17] Bass L, Clements P, Kazman R (2003). Software architecture in practice.

[18] Tekinerdogan B, Zdun U, Babar A. Software architecture. In: 10th European conference, ECSA 2016, Copenhagen, Denmark, November 28–December 2 vol. 9839. Springer, New York; 2014.

[19] ISO/IEC/IEEE: ISO/IEC/IEEE 42010 Systems and software engineering–architecture description. Technical report, ISO/IEC/IEEE 42010; 2011.

[20] Kassahun A. Aligning business processes and IT of multiple collaborating organisations. PhD thesis, Wageningen University; 2017. 10.18174/414988.

[21] Muller G. A reference architecture primer. Eindhoven, White paper. Eindhoven Univ. of Techn; 2012.

[22] US Dept. of Defence/Office of the DoD CIO: reference architecture description. Technical Report June, US Dept. of Defence/Office of the DoD CIO (2010). http://dodcio.defense.gov/Portals/0/Documents/DIEA/Ref_Archi_Description_Final_v1_18Jun10.pdf.

[23] Frandji B, Schot J, Joubert M, Soadyh I, Kilsdonk A (1994). The RICHE reference architecture. Med Inform.

[24] Wartena F, Muskens J, Schmitt L, Petković M. Continua: the reference architecture of a personal telehealth ecosystem. In: The 12th IEEE international conference on e-health networking, applications and services. IEEE; 2010. p. 1–6.

[25] Extreme: Healthcare reference architecture. Technical report, extreme networks; 2010. https://www.extremenetworks.com/resources/brochure/healthcare-reference-architecture/.

[26] Puscas K. Nationwide health information network exchange. Nationwide health information network: enterprise architecture overview. technical report; 2009.

[27] Brandara N. Connected health reference architecture. Technical report, WSO2; 2015. https://wso2.com/whitepapers/connected-health-reference-architecture/.

[28] Kipf OM. Reference architecture for healthcare—design concepts; 2020.

[29] Nictiz: Domain reference model for hospitals version 2. Technical report. Nationaal ICT Instituut in de Zorg; 2012. https://www.dragon1.com/downloads/12001A_Domain_Reference_hospitals_version_2_00.pdf.

[30] ATOS. IT Reference architecture for healthcare. Technical report, ATOS; 2011. https://atos.net/wp-content/uploads/2016/06/atos-itah-architecture-for-healthcare-whitepaper.pdf.

[31] Winter A, Brigl B, Funkat G, Häber A, Heller O, Wendt T (2007). 3LGM2-modeling to support management of health information systems. Int J Med Inform.

[32] Koksal O, Tekinerdogan B (2017). Obstacles in data distribution service middleware: a systematic review. Future Gen Comput Syst.

[33] van Geest M, Tekinerdogan B, Catal C (2020). Design of a reference architecture for developing smart warehouses in industry 4.0. Comput Ind.

[34] Tekinerdogan B, Aksit M. Classifying and evaluating architecture design methods. In: Software architectures and component technology. Springer: New York; 2002. p. 3–27.

[35] Clements P, Garlan D, Little R, Nord R, Stafford J, Bachmann F, Bass L, Ivers J, Merson P. Documenting software architectures: views and beyond. 2nd Ed. Addison-Wesley Professional; 2 ed (October 15, 2010), Boston 2010.

[36] Tummers J, Kassahun A, Tekinerdogan B. Reference architecture design for farm management information systems: a multi-case study approach. Precision Agriculture; 2020.

[37] Yan ED. Research about based-SOA agriculture management information system. In: International conference on information and automation (ICIA). IEEE; 2012. p. 78–82. 10.1109/ICInfA.2012.6246786.

[38] Murakami Y, Utomo S.K.T, Hosono K, Umezawa T, Osawa N. IFarm: development of cloud-based system of cultivation management for precision agriculture. In: IEEE 2nd global conference on consumer electronics (GCCE). IEEE; 2013. p. 233–234. 10.1109/GCCE.2013.6664809.

[39] Kassahun A, Hartog RJ, Tekinerdogan B (2016). Realizing chain-wide transparency in meat supply chains based on global standards and a reference architecture. Comput Electron Agric.

[40] Kitchenham B, Charters S. Guidelines for performing systematic literature reviews in software engineering. Technical report, Computer Science Department, Keele University and Department of Computer Science, University of Duram; 2007.

[41] Riahi S, Fischler I, Stuckey MI, Klassen PE, Chen J (2017). The value of electronic medical record implementation in mental health care: a case study. JMIR Med Inform.

[42] Lei J, Sockolow P, Guan P, Meng Q, Zhang J (2013). A comparison of electronic health records at two major Peking University Hospitals in China to United States meaningful use objectives. BMC Med Inform Decis Mak.

[43] Tierney WM, Sidle JE, Diero LO, Sudoi A, Kiplagat J, Macharia S, Shen C, Yeung A, Were MC, Slaven JE (2016). Assessing the impact of a primary care electronic medical record system in three Kenyan rural health centers. J Am Med Inform Assoc.

[44] Guo J, Iribarren S, Kapsandoy S, Perri S, Staggers N (2011). Usability evaluation of an electronic medication administration record (eMAR) application. Appl Clin Inform.

[45] Rajkovic P, Jankovic D, Milenkovic A (2013). Developing and deploying medical information systems for Serbian public healthcare: challenges, lessons learned and guidelines. Comput Sci Inf Syst.

[46] Tsavatewa C, Musa PF, Ramsingh I (2012). Integration of footprints information systems in palliative care: the case of Medical Center of Central Georgia. J Med Syst.

[47] Duke JD, Morea J, Mamlin B, Martin DK, Simonaitis L, Takesue BY, Dixon BE, Dexter PR (2014). Regenstrief Institute’s medical gopher: a next-generation homegrown electronic medical record system. Int J Med Inform.

[48] Sockolow PS, Weiner JP, Bowles KH, Abbott P, Lehmann HP (2011). Advice for decision makers based on an electronic health record evaluation at a program for all-inclusive care for elders site. Appl Clin Inform.

[49] Martinez W, Threatt AL, Rosenbloom ST, Wallston KA, Hickson GB, Elasy TA (2018). A patient-facing diabetes dashboard embedded in a patient web portal: design sprint and usability testing. JMIR Hum Fact.

[50] Tekinerdogan B, Öztürk K. Feature-driven design of SaaS architectures. In: Software engineering frameworks for the cloud computing paradigm. New York: Springer; 2013. p. 189–212.

[51] Kang KC, Cohen SG, Hess JA, Novak WE, Peterson AS. Feature-oriented domain analysis (FODA) feasibility study. Technical report. Carnegie-Mellon Univ Pittsburgh Pa Software Engineering Inst; 1990.

[52] Jonathan Mak WH, John Clarkson P (2017). Towards the design of resilient large-scale engineering systems. Proc CIRP.

[53] Kim CH, Weston RH, Hodgson A, Lee KH (2003). The complementary use of IDEF and UML modelling approaches. Comput Ind.

[54] Choubey MK (2011). IT infrastructure and management (For the GBTU and MMTU).

[55] Van Vliet H (1993). Software engineering: principles and practice.

[56] Sancar Gozukara S, Tekinerdogan B, Catal C. Obstacles of on-premise enterprise resource planning systems and solution directions. J Comput Inform Syst. 2020;1–12.

[57] Chellappa RK, Saraf N (2010). Alliances, rivalry, and firm performance in enterprise systems software markets: a social network approach. Inform Syst Res.

[58] Pollock N, Williams R (2011). Moving beyond the single site implementation study: how (and why) we should study the biography of packaged enterprise solutions. Inform Syst Res.

[59] Jahn F, Issler L, Winter A, Takabayashi K (2009). Comparing a Japanese and a German hospital information system. Methods Inf Med.

[60] Opara-Martins J, Sahandi R, Tian F (2016). Critical analysis of vendor lock-in and its impact on cloud computing migration: a business perspective. J Cloud Comput.

[61] Bender D, Sartipi K. Hl7 fhir: an agile and restful approach to healthcare information exchange. In: Proceedings of the 26th IEEE international symposium on computer-based medical systems. IEEE;2013. p. 326–331.

[62] World Organization of National Colleges, Academies, and Academic Associations of General Practitioners/Family Physicians. In: ICPC-2: international classification of primary care. Oxford: Oxford University Press; 1998.

[63] World Health Organization. International classification of diseases for mortality and morbidity statistics, 11th Revision; 2018. https://icd.who.int/browse11/l-m/en.

[64] ISO/IEC/IEEE: Life cycle processes—Requirements engineering. Technical report, ISO/IEC/IEEE; 2011.

[65] Open Group. ArchiMate 2.0 specification: Open Group Standard. Van Haren Publishing, ’s-Hertogenbosch, the Netherlands; 2012.

[66] Saha P (2008). Advances in government enterprise architecture.

[67] IEEE. IEEE 1471, Standard for describing the architecture of a “software-intensive system”. Technical report. Institute of Electrical and Electronic Engineers; 2000.

