# Supplementary material for: Designing a reference architecture for health information systems
Source: BMC Med Inform Decis Mak. 2021 Jul 8;21:210. doi: 10.1186/s12911-021-01570-2 (PMC8263849; doi:10.1186/s12911-021-01570-2)
Supplement: Supplementary file 1 — Additional file 1. Application architecture figures. [file 12911_2021_1570_MOESM1_ESM.pdf]

Appendix

May 18, 2021

Application architecture Figures

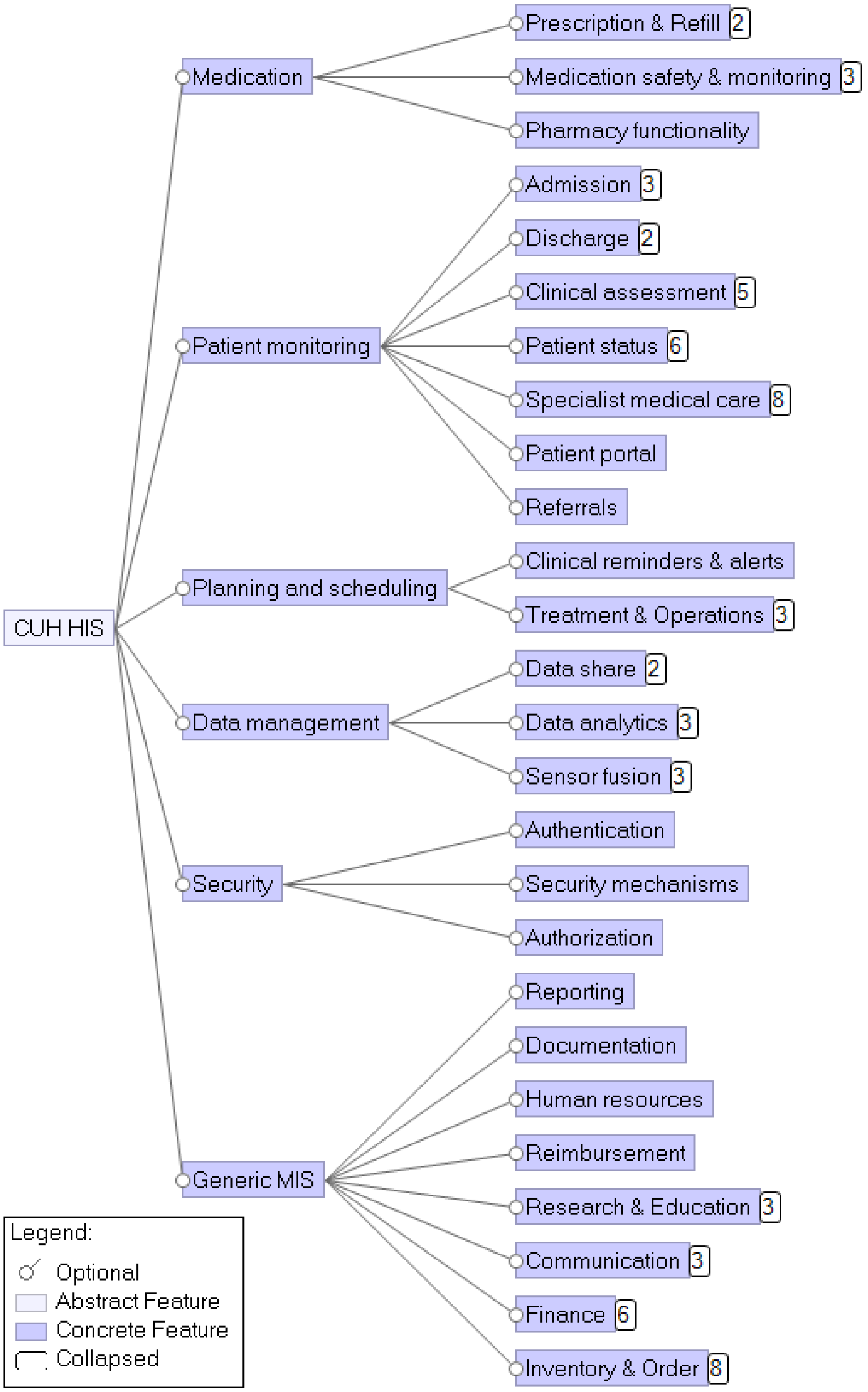

Figure 1: Downsized version of the feature model for the Chiba University Hospital based on Jahn et al. [59]. Numbers on the right hand side of the features represent the number of sub-features shown.

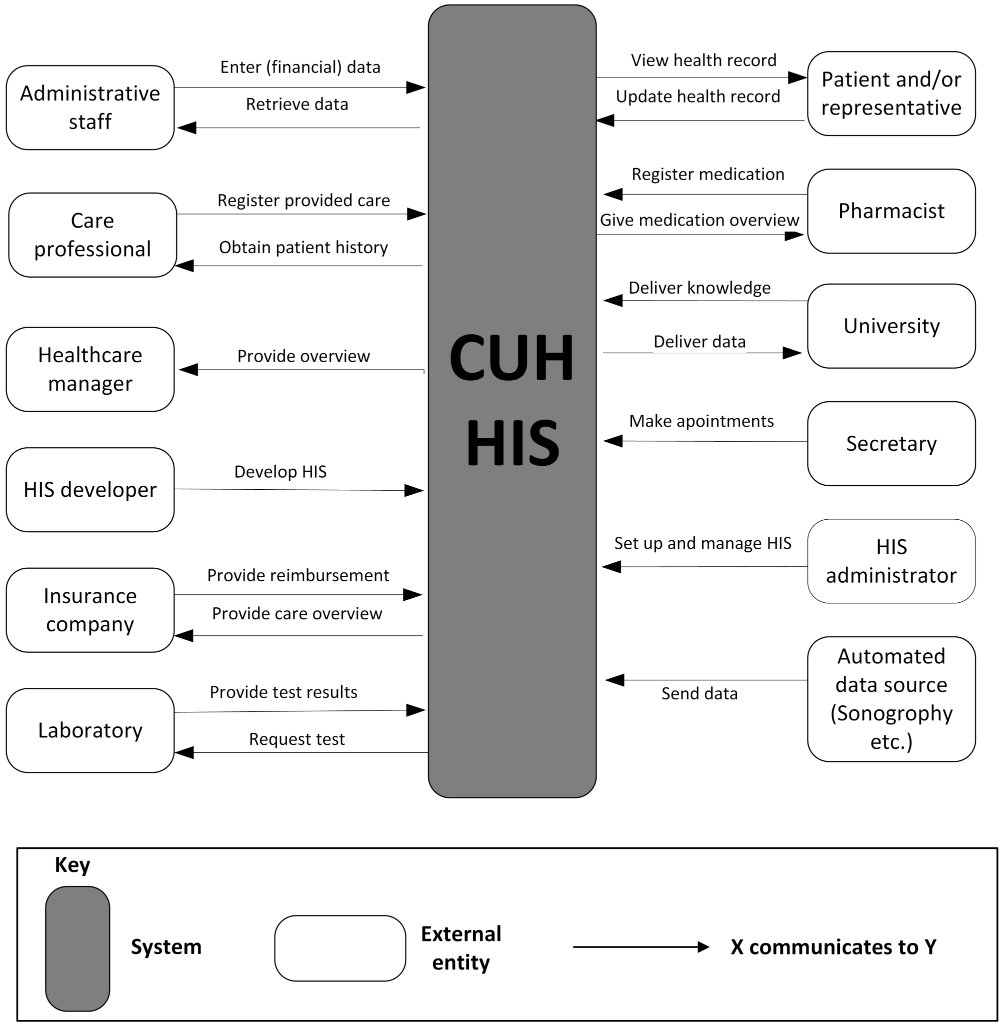

Figure 2: Context diagram for the Chiba University Hospital based on Jahn et al. [59]

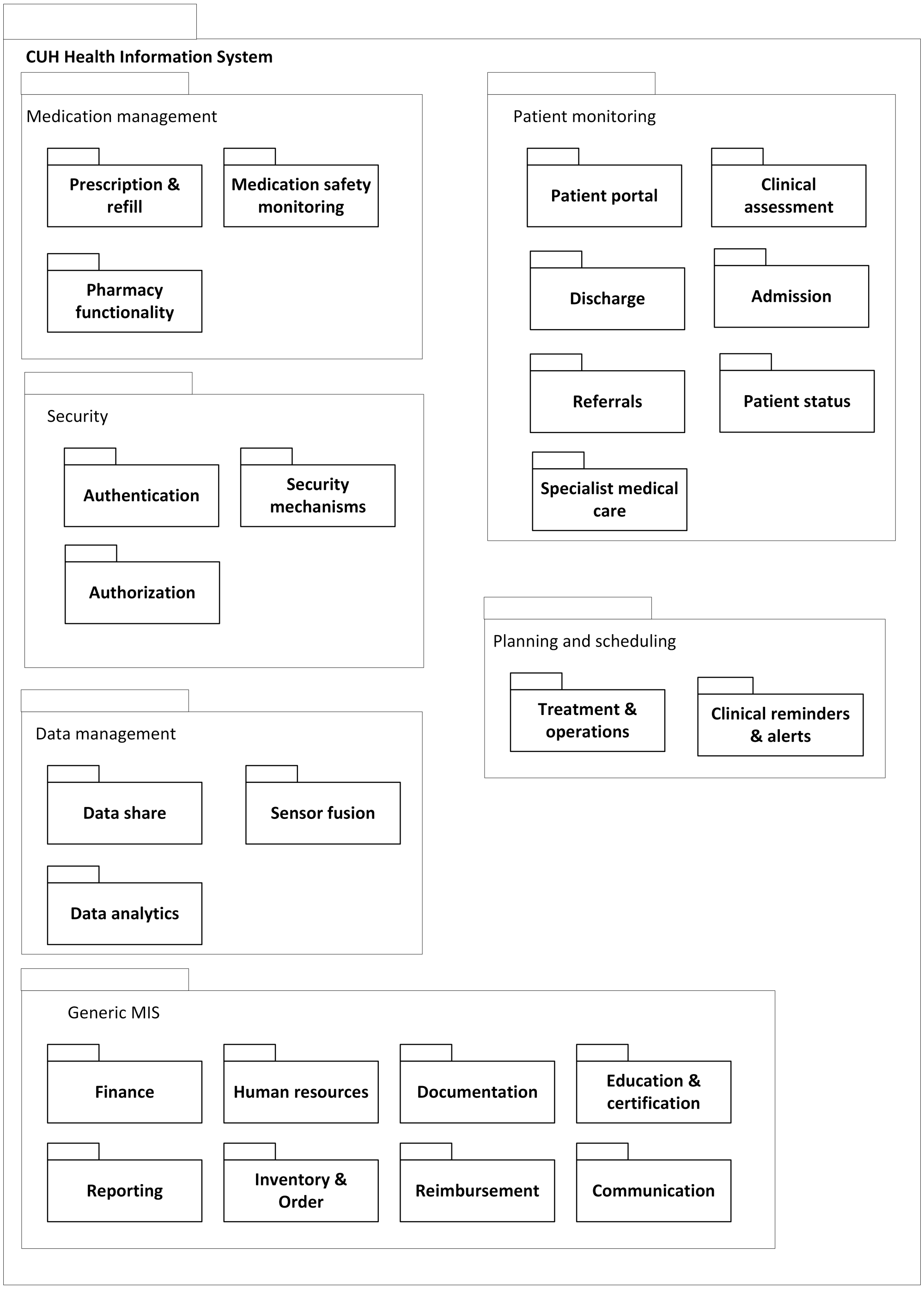

Figure 3: Downsized version of the decomposition view for the Chiba University Hospital based on Jahn et al. [59]
